# Tocilizumab Increases Serum Lipids but Does Not Increase Arteriosclerosis, As Measured by Intima-Media Thickness, in Patients With Rheumatoid Arthritis

**DOI:** 10.7759/cureus.76782

**Published:** 2025-01-02

**Authors:** Mayuko Moriyama, Yohko Murakawa, Masahiro Kondo, Mariko Taira, Yoshiko Sumita, Manabu Honda, Kunihiro Ichinose

**Affiliations:** 1 Department of Rheumatology, Shimane University Faculty of Medicine, Izumo, JPN; 2 Department of Internal Medicine III, Shimane University Faculty of Medicine, Izumo, JPN; 3 Center of Community Medical Policy, Shimane University Faculty of Medicine, Izumo, JPN; 4 Department of Rheumatology, Shimene University Faculty of Medicine, Izumo, JPN; 5 Department of Internal Medicine, Izumo Citizens' Rehabilitation Hospital, Izumo, JPN

**Keywords:** arteriosclerosis, carotid intima-media thickness, high-resolution b-mode ultrasonography, rheumatoid arthritis, tocilizumab

## Abstract

Objectives

Patients with rheumatoid arthritis (RA) have a high incidence of arteriosclerotic disease. These are partly attributed to high levels of C-reactive protein (CRP) and inflammatory cytokines, such as tumor necrosis factor-alpha (TNF-α) and interleukin (IL)-6. Tocilizumab (TCZ) is an IL-6 receptor antagonist that lowers CRP levels by directly blocking IL-6 signaling. Tocilizumab has been reported to increase serum lipid levels. However, its effect on arteriosclerosis remains unclear. Therefore, we investigated the effect of TCZ on arteriosclerosis in patients with RA.

Methods

Eighteen Japanese patients with RA who were administered TCZ were included and assessed at baseline and six and 12 months. The Disease Activity Score-28 with erythrocyte sedimentation rate (DAS28-ESR), CRP, total cholesterol (TC), low-density lipoprotein cholesterol (LDL-C), high-density lipoprotein cholesterol (HDL-C), and carotid intima-media thickness (IMT) were measured and analyzed using high-resolution B-mode ultrasonography. Additionally, the effects of concurrent statin administration on any changes in IMT were evaluated.

Results

From baseline to six or 12 months, TCZ decreased CRP (P < 0.0005) and DAS28-ESR (P < 0.0005) significantly, whereas TC, LDL-C, and HDL-C levels increased significantly (P < 0.005). However, there was no change in the LDL-C/HDL-C ratio (P = 0.821 at six months and P = 0.168 at 12 months), and carotid IMT (P = 0.6874 at six months and P = 0.6951 at 12 months). Comparison of percentage changes in mean IMT revealed no statistical differences between the patient groups with or without statin administration (P = 0.7208 at six months, P = 0.5928 at 12 months).

Conclusions

According to the 12-month observation data, no significant association was detected between TCZ use and IMT changes in patients with RA, despite its effects on serum lipids. Further long-term studies are needed to confirm that IL-6 receptor blockers have cardiovascular effects.

## Introduction

Rheumatoid arthritis (RA) is a chronic inflammatory disease that leads to progressive joint deformity, disability, and premature death. Most studies on mortality in patients with RA have indicated a greater risk of death than that in an age-matched general population. Cardiovascular disease (CVD) is the primary cause of premature death in RA patients [1, 2]. Thus, targeting a reduction in arteriosclerosis and CVD should be considered a significant issue in the treatment of patients with RA.

The mechanism underlying the higher prevalence of CVD in patients with RA may be explained by the close link between chronic inflammation and atherosclerosis [3]. Tumor necrosis factor (TNF) inhibitors decrease the risk of cardiovascular mortality in patients with RA by ameliorating inflammation [4]. Furthermore, interleukin (IL)-6 is a common pathological factor in RA [5] and atherosclerosis [6].

Tocilizumab (TCZ) is an IL-6 receptor antagonist that lowers C-reactive protein (CRP) through direct blockade of IL-6 signaling [7]. As inflammation contributes to the progression of atherosclerosis, TCZ treatment may be beneficial in protecting patients with RA from cardiovascular events, similar to tumor necrosis factor-alpha (TNF-α) blockers. However, TCZ has been reported to increase serum lipids [7]. Therefore, it is important to examine their effects on arteriosclerosis. In the present novel study, we examined the carotid intima-media thickness (IMT), which is increasingly used as a surrogate marker for atherosclerosis and a predictor of future cardiovascular events in healthy individuals [8].

## Materials and methods

Patients

The study population was recruited from consecutive patients at Shimane University Hospital, Izumo, Japan. All patients who introduced TCZ into routine clinical practice and provided informed consent for the study were enrolled. Eighteen Japanese patients with RA were examined for carotid IMT at all study points. The patients were studied from January 26, 2009 to March 2013.

Disease assessments and laboratory tests

Disease activity was measured using the composite index Disease Activity Score-28 with erythrocyte sedimentation rate (DAS28-ESR) and calculated according to the following formula: The ESR and CRP were measured using routine methods. Serum total cholesterol (TC), high-density lipoprotein cholesterol (HDL-C), and low-density lipoprotein cholesterol (LDL-C) levels were determined using an autoanalyzer.

Ultrasonographic determination of the carotid IMT

The carotid IMT was measured using high-resolution B-mode ultrasonography. Briefly, patients were placed supine for ultrasonographic examination of the bilateral common carotid arteries. The common carotid arteries were scanned at the bifurcation levels on the right and left sides. The IMT was measured at the far wall of the arteries at sites of greatest arterial thickness on diffuse and continuous projection. Subsequently, the greatest distance between the lumen-intima interface and the media-adventitia interface in areas without atherosclerotic plaques (localized lesions > 2.0 mm in thickness) was determined. The IMT-Cmax was defined as the maximum intima-media thickness of the common carotid artery. Furthermore, changes in mean IMT were analyzed as percentage changes from baseline IMT.

Statistical analyses

The Wilcoxon test was used to compare each parameter between baseline and at six and 12 months. Mann-Whitney U tests were used to compare statin, methotrexate (MTX), or prednisolone patients with those who did not. Pearson’s correlation coefficient was used to ascertain whether correlations existed between any two parameters. A P-value of < 0.05 indicated statistical significance.

Study protocol approval

The study was approved by the ethics committee of Shimane University Faculty of Medicine (approval no. 416 dated 29 September 2008) and performed in accordance with the Declaration of Helsinki. Written informed consent was obtained from all participants.

## Results

Clinical characteristics of the enrolled patients

Eighteen Japanese RA patients were examined at baseline, six months, and 12 months after starting TCZ therapy. Table 1 shows the clinical characteristics of the patients at baseline.　

**Table 1:** Clinical characteristics of the patients with rheumatoid arthritis at baseline n: number; SD: standard deviation; DAS28-ESR: Disease Activity Score-28 with erythrocyte sedimentation rate, IMT: intima-media thickness

Clinical characteristics at baseline	Total n = 18
Female, n (%)	12 (66.7)
Age, years ± SD	65.1 ± 8.59
Disease duration, months ± SD	65.0 ± 57.4
Smoking, n (%)	6 (33.3)
Hypertension, n (%)	6 (33.3)
Diabetes mellitus, n (%)	7 (38.9)
History of ischemic heart disease, n (%)	1 (0.05)
Previous use of biologics, n (%)	5 (27.8)
Statin use, n (%)	8 (44.4)
Methotrexate use, n (%)	6 (33.3)
Methotrexate dose (n = 6), mg/week ± SD	9.33± 2.48
Prednisolone use, n (%)	15 (83.3)
Prednisolone dose, mg/day	6.70 ± 3.99
DAS28-ESR, score ± SD	5.04 ± 1.34
Carotid IMT-Cmax, mm ± SD	1.064 ± 0.373

Disease activity and clinical laboratory findings

**Figure 1:**
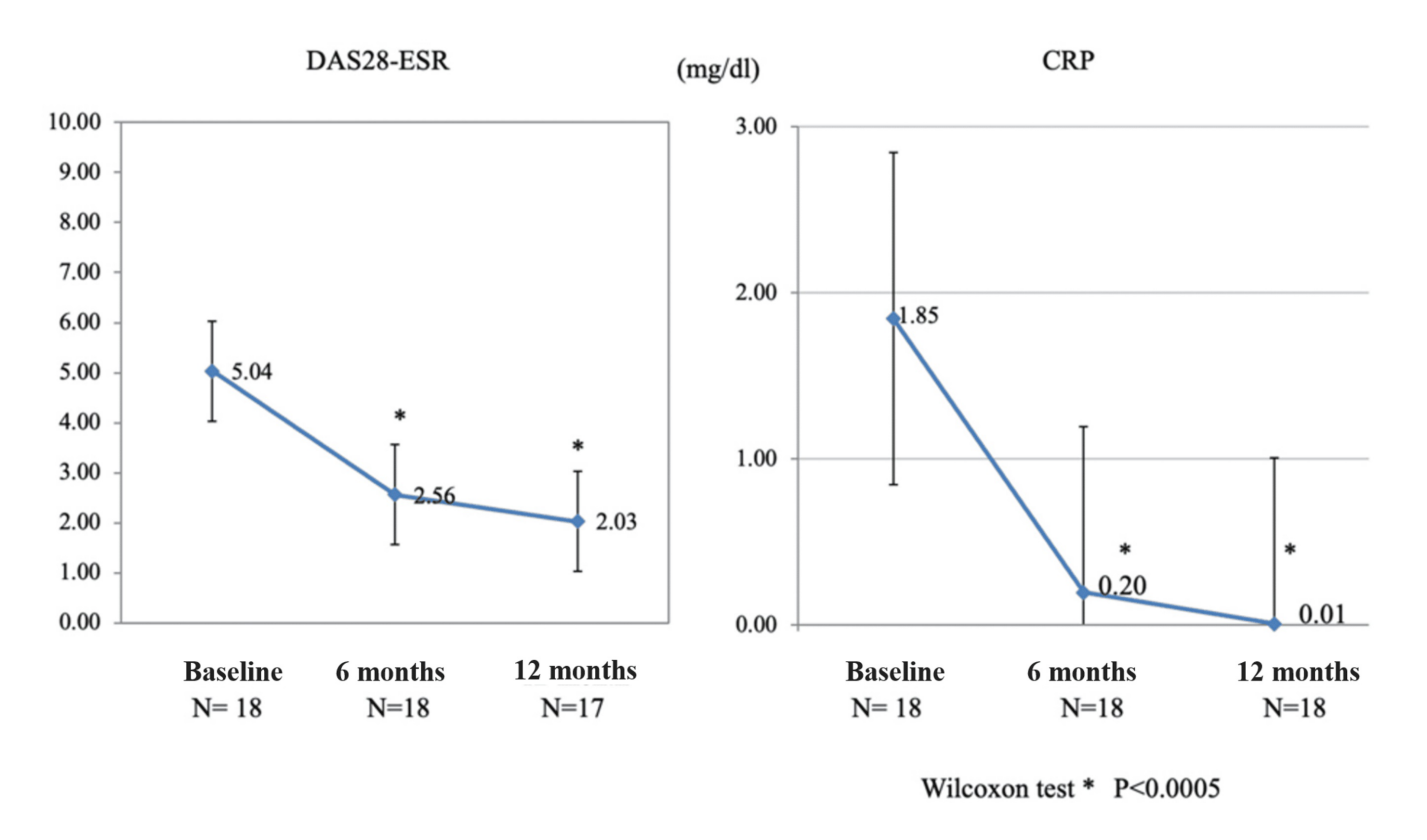
Treatment with tocilizumab significantly improves the DAS28-ESR and CRP levels in patients with rheumatoid arthritis The Wilcoxon test showed a significant decrease from baseline to six or 12 months for both DAS28-ESR and CRP levels (P < 0.0001). The data are expressed as mean ± standard deviation; * represents P < 0.0005. DAS28-ESR: Disease Activity Score-28 with erythrocyte sedimentation rate; CRP: C-reactive protein; N: number of patients

Tocilizumab was administered at a dose of 8 mg/kg every four weeks. The CRP decreased significantly, from 1.85±2.12 mg/dL at baseline to 0.20±0.76 mg/dL (P < 0.0005) at six months and 0.01±0.01 mg/dL (P < 0.0005) at 12 months. Additionally, the DAS28-ESR score improved from 5.04±1.34 at baseline to 2.56±1.23 (P < 0.0005) at six months and to 2.03±1.26 (P < 0.0001) at 12 months (Figure 1). 

**Figure 2:**
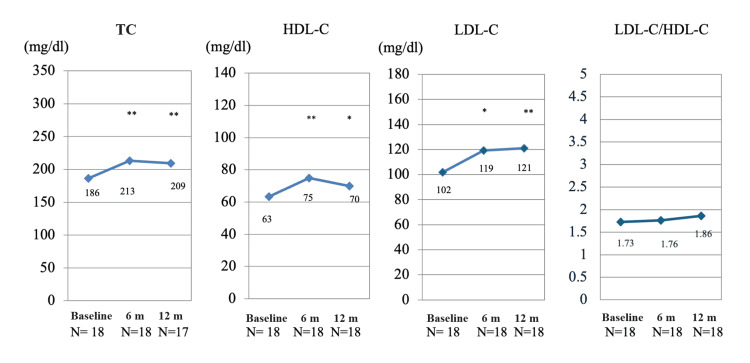
Tocilizumab treatment increases serum lipids but not the LDL/HDL ratio in rheumatoid arthritis patients The Wilcoxon test shows a significant increase in serum total cholesterol (TC), high-density lipoprotein cholesterol (HDL-C), and low-density lipoprotein cholesterol (LDL-C) levels from baseline to six and 12 months. However, there was no significant difference in the LDL-C/HDL-C ratio from baseline to six months (P = 0.821) or from baseline to 12 months (P = 0.168). A P-value of < 0.05 (*) or < 0.005 (**) indicated statistical significance. N: number of patients; m: months

In contrast, TCZ therapy significantly increased serum lipid levels (Figure 2). The level of TC increased from 186±35 mg/dL to 213±32 mg/dL (P = 0.0075) at six months and to 209±34 mg/dL (P = 0.0098) at 12 months. Both HDL-C and LDL-C significantly increased from 63±18 mg/dl to 75±21 mg/dl (P = 0.0097) and 70±18 mg/dl (P = 0.0174) and from 102±25 mg/dl to 119±27 mg/dl (P = 0.0121) and 121±29 mg/dl (P = 0.0011), respectively (Figure 2). Thus, TCZ increased not only HDL-C but also LDL-C. To confirm the effect of statins, patients were divided into those who had taken statins for three months before the start of the study and those who had not taken statins. Although the trends varied from patient to patient due to limitations of the small number of participants, serum lipids tended to increase at six months regardless of whether they were taking statins. At 12 months, LDL-C tended to increase in patients not taking statins, and two patients were newly administered statins during the study (one patient took statins from weeks eight to 36, and the other took statins after week 20). None of the patients who had been taking statins before the start of the study strengthened their treatment for dyslipidemia, and lipid levels did not increase at 12 months compared to six months, although they increased from baseline (data not shown). The LDL-C/HDL-C ratio was not significantly different from baseline at six months (P = 0.821) or baseline at 12 months (P = 0.168) but is more closely related to arteriosclerosis (Figure 2).

Changes in carotid IMT during TCZ therapy

**Figure 3:**
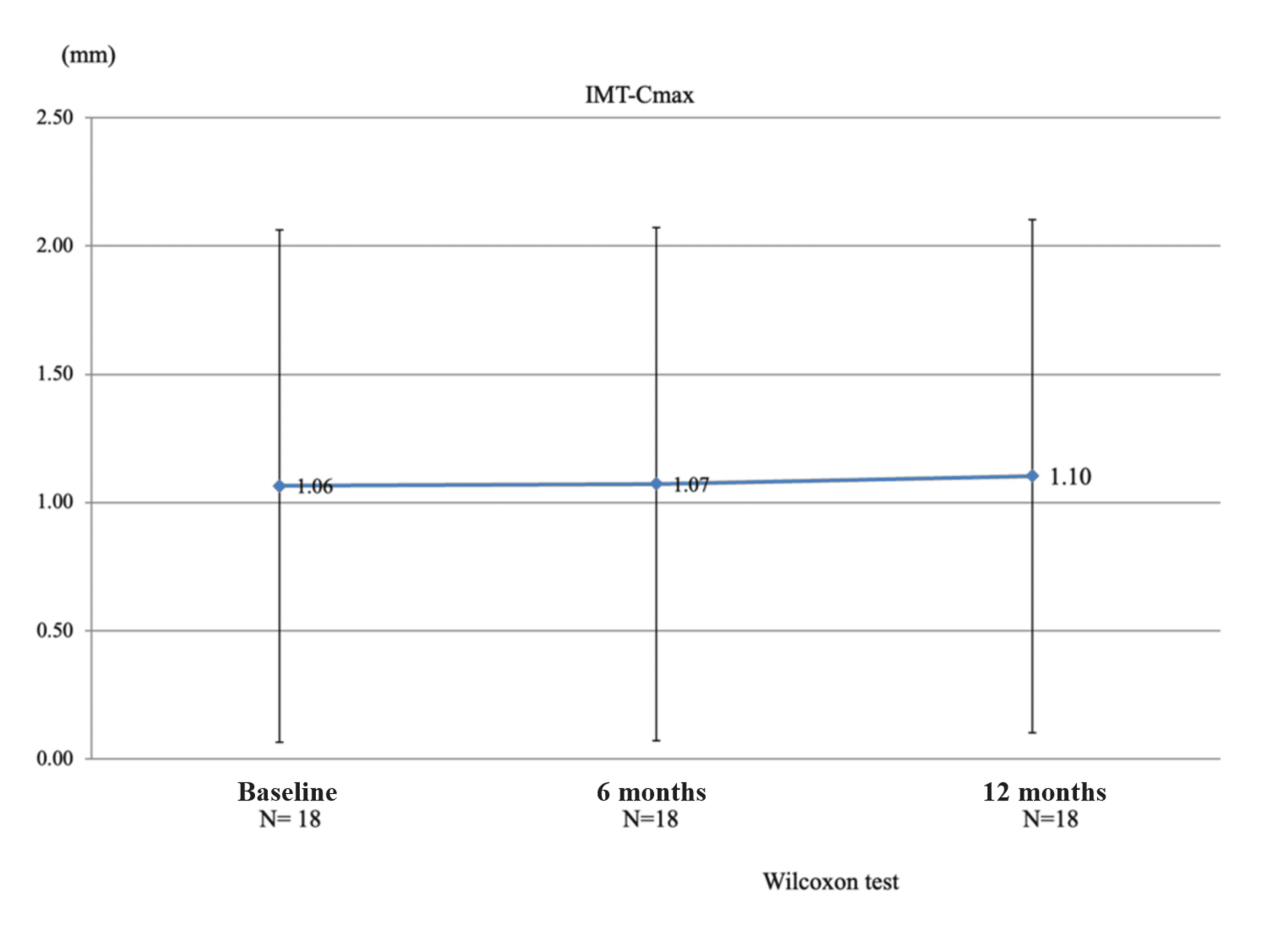
Tocilizumab (TCZ) did not promote carotid intima-media thickness (IMT) during a 12-month treatment period The bilateral carotid IMT in patients with rheumatoid arthritis (RA) is measured using B-mode ultrasonography at baseline and six and 12 months during TCZ therapy. The Wilcoxon test shows no significant increase in carotid IMT in patients with RA (P = 0.6874 at six months, P = 0.5928 at 12 months). The data are expressed as mean ± standard deviation. N: number of patients

We investigated the carotid IMT using B-mode ultrasonography. Carotid IMT in the patients was 1.01±0.32 mm at baseline, 1.06±0.37 mm at baseline, 1.07±0.38 mm (P = 0.6874) at six months, and 1.10±0.49 mm (P = 0.6951) (Figure 3). No significant change was observed in carotid IMT, and no cardiovascular events occurred during the study period.

For three months before the start of the study, eight of 18 patients received statins that influence lipid metabolism and arteriosclerosis. Thus, we analyzed IMT in two groups: those taking versus not taking statins. Changes in the mean IMT in the eight patients taking statins versus the 10 patients not taking statins were compared using the Mann-Whitney U-test. Statin administration tended to cause changes; however, there was no statistically significant difference between the two groups (P = 0.7208 at six months and P = 0.5928 at 12 months).

Additionally, there was no significant difference in IMT between patients with high and low disease activity at baseline or between those taking prednisolone or MTX.

## Discussion

Aside from arteriosclerosis, RA is a risk factor for CVD, such as ischemic heart disease and cerebrovascular accidents [1, 2]. The risk caused by RA is compatible with that caused by type 2 diabetes mellitus [9]. However, arteriosclerosis development in RA is related to chronic inflammation, apolipoproteins, and anti-phosphorylcholine IgM presence [10]. Several recent studies investigated the effects of RA therapy on mortality, CVD, and arteriosclerosis [4]. Van Doornum et al. reported that six weeks of treatment with TNF antagonists did not improve arterial stiffness despite significant reductions in synovitis and inflammatory markers in 14 patients with RA [11]. However, the study period may have been too short to detect any effects on arterial stiffness. 

Most studies on TNF inhibitors have demonstrated decreased CVD risk in RA patients. Infliximab improves endothelial function in RA [12, 13], partly due to elevated serum levels of adiponectin [12], which were previously shown to suppress the development of atherosclerosis. Furthermore, TNF inhibitors reduce the effects of TNF on CVD, such as dyslipidemia, insulin resistance, and adhesion molecules, which lead to inflammation and fatty streaks in the vascular walls [14]. Moreover, TNF inhibitors may reduce thrombus formation and ischemic damage in CVD [14].

In recent years, TCZ has proved to be effective in treating RA [15], and it is used worldwide. In the present study, we demonstrated that TCZ induced significant reductions in inflammatory markers and disease activity in all patients with RA but significantly elevated serum lipid markers such as TC, LDL-C, and HDL-C [7]. Therefore, it is crucial to investigate how TCZ affects atherosclerosis because IL-6 and CRP are linked to CVD, even in healthy males [16].

We investigated the changes in carotid IMT, which is thought to be caused by dyslipidemia and inflammation in RA [10, 17]. It is a predictor of CVD [8] and was previously established to be an arteriosclerotic marker [8]. A recent study observing changes in carotid IMT in patients with dyslipidemia measured carotid IMT at weeks 26, 52, 78, and 104 in the rosuvastatin and placebo groups. Differences were observed from week 26, and the difference in the annual rate of change was significant (P = 0.020). In the rosuvastatin group, there seemed to be a difference in the annual change in carotid IMT between participants who showed changes in LDL-C below the mean value and those who showed changes above the mean value [18]. Therefore, it is important not to increase lipid levels, especially LDL-C. In our study, TCZ caused an increase in serum lipids, but no significant changes were observed in carotid IMT. In this study, a significant increase in serum lipids was observed during 12 months of TCZ therapy, but no significant increase in IMT was observed with or without statins. This result may be based on the fact that TCZ increased not only LDL-C but also HDL-C, and the LDL-C/HDL-C ratio did not change in RA patients. Therefore, it is interpreted that TCZ is unlikely to cause atherosclerosis, although it increases serum lipids.　In addition, although an increase in serum lipids was observed, the increase was limited in the Japanese subjects of the study, and it may not have been enough to cause arteriosclerosis. Moreover, this may be because the suppression of inflammation by TCZ inhibits the progression of atherosclerosis in arteries. Hence, our findings are in line with those of recent studies on arteriosclerosis that evaluated endothelial function using flow-mediated dilatation (FMD), pulse wave velocity (PWV), and cardio-ankle vascular index (CAVI) over six months of TCZ therapy [19,20]. Additionally, our study findings are similar to a study of 38 patients with RA treated with non-TNF antagonists, seven of whom were treated with TCZ [21]. In adipocytes, IL-6 reduces the expression and secretion [22]. Similar to TNF inhibitors, blocking IL-6 may enhance adiponectin secretion. 

Patients with RA have lower HDL concentrations than control participants because excess IL-6 in inflammatory situations such as RA lowers blood lipid levels [23]. As a result, by inhibiting the IL-6 pathway, TCZ promotes the partial restoration of apolipoprotein synthesis and raises blood levels of TC [24]. Serum LDL-C and HDL-C levels may exhibit similarity to the intrinsic values that patients would generally possess based on their lifestyle and diet without excess IL-6 due to TCZ treatment. Although TCZ increases blood lipid concentrations, the results of the 12-month observation period suggest that it may not cause atherosclerosis in patients with RA.

Previously, TNF inhibitors and abatacept were reported to have a lower risk of CVD than conventional synthetic disease-modifying antirheumatic drugs (csDMARDs) [25]. Although no significant reduction in CVD risk was observed, a trend was observed for TCZ in this study [25]. In contrast, a recent study on the therapeutic effect of IL-6R inhibitors in different types of CVD was reported based on genome-wide association study (GWAS) summary statistics using a Mendelian randomization (MR) approach and found that IL-6R inhibitors had no effect on hypertension, atrial fibrillation, or heart failure but had a significant effect on coronary heart disease and myocardial infarction [26]. Genetically predicted IL-6R inhibitors were associated with a protective effect against coronary heart disease and myocardial infarction [26].

The strength of our study is that the effects of TCZ on blood vessels were evaluated using carotid IMT following a time series. Given the minimal impact of TCZ administration on carotid IMT, the need to correct dyslipidemia should be discussed.

This study has several limitations. First, the sample size was relatively small; hence, additional patients should be included for a more detailed analysis. Second, this study was conducted in Japanese patients with RA; therefore, the generalizability of the findings to other populations may be limited. Third, the observation period was short (up to 12 months), and the long-term effects of TCZ on blood vessels are yet to be fully observed. Fourth, the risk of CVD and chronic kidney disease (CKD) in patients with RA are interrelated, and the present study did not exclude the influence of CKD. Further research is required to determine whether IL-6 receptor blockers have angioprotective effects. Additionally, we should carefully monitor and treat lipid abnormalities.

## Conclusions

According to the 12-month observational data, no significant association was detected between the use of IL-6 receptor antagonists in RA patients and changes in IMT, despite the effect on serum lipids. One reason may be that IL-6 receptor antagonists increase not only LDL-C but also HDL-C, and the LDL-C/HDL-C ratio did not increase significantly compared to baseline. Careful consideration may be needed regarding the need to introduce statins for lipid abnormalities when using IL-6 receptor blockers. Further long-term studies are needed to confirm that IL-6 receptor blockers have a vascular protective effect.
